# Dose-dependence of radiotherapy-induced changes in serum levels of choline-containing phospholipids; the importance of lower doses delivered to large volumes of normal tissues

**DOI:** 10.1007/s00066-021-01802-4

**Published:** 2021-06-29

**Authors:** Karol Jelonek, Aleksandra Krzywon, Katarzyna Papaj, Pawel Polanowski, Krzysztof Szczepanik, Krzysztof Skladowski, Piotr Widlak

**Affiliations:** 1 Maria Sklodowska-Curie National Research Institute of Oncology, Gliwice Branch, Wybrzeze Armii Krajowej 15, 44-102 Gliwice, Poland; 2 Biotechnology Centre, Silesian University of Technology, Krzywoustego 8, 44-100 Gliwice, Poland

**Keywords:** Head and neck cancer, Mass spectrometry, Lipidomics, Ionizing radiation, Radiation response

## Abstract

**Background:**

Conformal radiotherapy is a primary treatment in head and neck cancer, which putative adverse effects depend on relatively low doses of radiation delivered to increased volumes of normal tissues. Systemic effects of such treatment include radiation-induced changes in serum lipid profile, yet dose- and volume-dependence of these changes remain to be established.

**Methods:**

Here we analyzed levels of choline-containing phospholipids in serum samples collected consecutively during the radiotherapy used as the only treatment modality. The liquid chromatography–mass spectrometry (LC-MS) approach applied in the study enabled the detection and quantitation of 151 phospholipids, including (lyso)phosphatidylcholines and sphingomyelins.

**Results:**

No statistically significant differences were found in the pretreatment samples from patients with different locations and stages of cancer. To compensate for potential differences between schemes of radiotherapy, the biologically effective doses were calculated and used in the search of correlations with specific lipid levels. We found that the levels of several phospholipids depended on the maximum dose delivered to the gross tumor volume and total radiation energy absorbed by the patient’s body. Increased doses correlated with increased levels of sphingomyelins and reduced levels of phosphatidylcholines. Furthermore, we observed several phospholipids whose serum levels correlated with the degree of acute radiation toxicity.

**Conclusion:**

Noteworthy, serum phospholipid levels were associated mainly with volumes of normal tissues irradiated with relatively low doses (i.e., total accumulated dose 20 Gy), which indicated the importance of such effects on the systemic response of the patient’s organism to intensity-modulated radiotherapy (IMRT).

**Supplementary Information:**

The online version of this article (10.1007/s00066-021-01802-4) contains supplementary material, which is available to authorized users.

## Introduction

Head and neck squamous cell carcinoma (HNSCC) involves different squamous cell carcinomas located in the larynx, pharynx, oral cavity, and tongue, i.e., organs that play crucial roles in respiratory, nutritional, social, and communicative functions. HNSCC is the sixth most common malignancy and accounts for approximately 6% of all cancer cases worldwide [1]. The primary treatment for HNSCC is surgery and/or radiotherapy (RT) applied alone or in combination with chemotherapy [2]. Currently, RT is delivered using techniques of conformal radiation therapy, including intensity-modulated radiotherapy (IMRT), where a high dose better conforms to the tumor shape, enabling a reduction of the dose delivered to adjacent critical organs [3]. However, a potential drawback of IMRT is an exposure of a large volume of normal tissues to low/medium doses. Hence, this approach may still increase the risk of undesirable adverse effects in normal tissues, which may not only reduce the patients’ quality of life [4] but also lead to unplanned therapy interruptions [5]. Importantly, in HNSCC a one-day gap in RT could decrease the local control rate by 1.4%, while a gap of one week is correlated with an absolute reduction in local control rates of 10–12% [6]. Therefore, hypothetical molecular markers for the monitoring of individual response to radiation might significantly improve the quality of HNSCC treatment.

The response to RT was already observed as the systemic effect in body fluids at all “omics” levels, including genomics, proteomics, and metabolomics [7–12]. Lipidomics is one of the most complex areas of metabolomics and it is dealing with dynamic changes of cellular lipids and their derivatives [13]. Glycerophospholipids (GP) and sphingolipids (SL) that include a choline group have essential structural and signaling functions [14, 15]. These classes of lipids were already shown to be affected in response to ionizing radiation, both local body RT in humans [16] and whole-body irradiation in rodents [17]. Our previous study on lipid mass profiling documented that serum levels of different phospholipids were affected in samples of HNSCC patients treated with IMRT using the continuous accelerated irradiation (CAIR) scheme. Moreover, the analysis based on the comparison of pre-RT and post-RT samples revealed that changes in levels of several compounds (putatively choline-containing phospholipids) were associated not only with a maximum dose delivered to the tumor target but also with a volume of tissues irradiated with lower doses [18]. Here we plan to further extend this observation, aiming to identify specific choline-containing lipids affected by radiation during RT and to reveal the dependence of RT-related effects on a radiation dose and volume of irradiated tissues using a series of serum samples collected through the whole treatment.

## Materials and methods

### Patient group and material collection

The study involved 45 patients (all Caucasians) with locally advanced HNSCC (no distant metastases) who received RT alone and did not undergo any other treatment (surgery or chemotherapy). The patients were treated with 6 different IMRT plans where a total dose was ranging from 51 Gy to 72 Gy, a fraction dose was ranging from 1.8 Gy to 3 Gy and a number of fractions was ranging from 17 to 40. Moreover, pretreatment samples of 53 patients with HNSCC were included; pre-RT and within-RT samples came from a partially overlapped group of patients because some pre-RT samples in the IMRT group were missed while only pretreatment samples of patients who received concurrent chemoradiotherapy were included to avoid the potential confounding effect of chemotherapy. Clinical characteristics of the enrolled patients and treatment details are presented in Table 1. Treatment-related regression of tumor was performed at least once a week or more often, if required. This procedure consisted of the following: a medical examination—palpation and laryngological examination with an assessment of tumor and lymph nodes regression, nasofiberoscopy (after physician decision), and blood tests. Acute toxicity (acute radiation sequel, ARS) was evaluated weekly during the treatment using a multiparametric scoring system based on morphological and functional factors [19]. The ARS system is based on the existing rules of the Common Toxicity Criteria of Adverse Event (CTCAE) scale and considers all symptoms related to the irradiated fields and affected functions collectively. Blood samples were collected once a week during radiotherapy (3–8 samples, depending on a patient; 249 samples from 45 patients) and before the start of any treatment (53 samples). Blood samples (5 ml) were collected into BD vacutainer tubes and incubated for 30 min at room temperature. Next, they were centrifuged at 1000 × *g* for 10 min. The resulting sera were portioned, then frozen, and stored at −80 °C.

**Table 1:** Characteristics of the patient group

Samples	Pre-RT	Within-RT
Number of patients	53	45
Number of samples	53	249
Age, years (median)	40–75 (57)	46–77 (62)
Sex, male/female	40/13	34/11
**Tumor location**	*n*	*n*
Larynx	27	31
Pharynx	26	11
Oral and nasal cavity	–	3
**Tumor size (T)**	*n*	*n*
T1	5	5
T2	22	29
T3	16	8
T4	10	3
**Lymph node status (N)**	*n*	*n*
N0	27	39
N1	4	3
N2	20	3
N3	2	0
**IMRT plan**		*n*
1	1.8 Gy/fraction; 38–40 fractions; 68.4–72 Gy total dose	23
2	2 Gy/fraction; 30–35 fractions; 60–70 Gy total dose	5
3	2.2 Gy/fraction; 30 fractions; 66 Gy total dose	5
4	2.25 Gy/fraction; 27 fractions; 60.75 Gy total dose	1
5	2.5 Gy/fraction; 24–25 fractions; 60–62.5 Gy total dose	7
6	3 Gy/fraction; 17 fractions; 51 Gy total dose	4

*RT* radiotherapy, *IMRT* intensity-modulated radiotherapy, *Gy* Gray

### Recalculation of received doses to BED

First, we determined irradiated volumes for selected isodoses starting from 2 Gy, then every 5 Gy from 5–30 Gy, and every 10 Gy from 40–70 Gy. To account for differences in biological effectiveness between applied treatment plans isodoses received by the treated HNSCC patients were recalculated to biologically effective doses (BED). BED is a measure of the biological dose delivered by a particular combination of dose per fraction and total dose to a particular tissue characterized by a specific α/β ratio [20]. Most of the volume irradiated by IMRT was related to healthy tissue; therefore the α/β ratio was kept at 3 for all calculations. Isodoses from each completed treatment plan were first adjusted to BED and then used to determine a BED-based dose–volume histogram (DVH). Since DVH reflects the total dose received during the whole treatment, we also adjusted DVH to individual samples based on how many fractions were received by the patient before the collection of the particular sample. We assumed the linear buildup of each isodose through the whole treatment. Therefore isodoses that were obtained at the end of treatment were divided by the total number of fractions to receive the isodose after the first fraction then further multiplied to obtain the isodose specific for a given time point. Finally, we obtained BED-based DVH for every individual sample (249 DVHs). Similarly, the maximum gross tumor volume (GTV) dose was adjusted to BED and calculated for each fraction assuming the linear buildup model. Examples of recalculation of physical doses to BED are presented in the Supplementary File Table S1. The descriptive statistics of radiation parameters are presented in the Supplementary File Table S2.

### Sample preparation and extraction of phospholipids

Each serum sample (10 μL) was complemented with LPC 17:1 (3.82 ng; Avanti Polar Lipids, Inc., Alabaster, AL, USA) and PC 17:0-14:1 (52 ng; Avanti Polar Lipids, Inc., Alabaster, AL, USA) standards before lipid extraction. Extraction of a lipid fraction was performed according to a modified Folch method [21]. In brief, 10 μL of serum was mixed with 350 μL of 1:1 methanol/chloroform mixture (v/v) containing antioxidants: 0.01% (w/v) 2,6-di-tert-butyl-4-methylphenol (Merck SA, Darmstadt, Germany) and 0.005% (w/v) retinol (Merck SA, Darmstadt, Germany). Then, 100 μL of water was added. The mixture was vortexed and incubated for 30 min at 4 °C and then centrifuged (5 min, 15,000 × *g*). The chloroform phase (the bottom one) was kept and stored at −80 °C, until performing mass spectrometry analysis (within one week).

### LC-MS analysis of phospholipids

The chloroform phase from the extraction step was diluted ten times with acetonitrile prior liquid chromatography–mass spectrometry (LC-MS) analysis. An aliquot of 8 μl of the resulting mixture was separated by Agilent 1290 Infinity LC (Agilent Technologies) using a 2.1 × 100 mm ACQUITY UPLC BEH HILIC column (Waters) with the flow rate of 250 μL/min. The chromatography was performed using 95:5 acetonitrile/water (solvent A) and 50:50 acetonitrile/water (solvent B), both with 10 mM ammonium acetate at pH 8.0; gradient of solvent B from 0% to 30% was applied within 15 min, and resulting fractions were analyzed online using QTOF 6540 mass spectrometer (Agilent) in the positive electrospray ionization mode. Spectra were preprocessed by peak picking and alignment, and then peak abundances (area under the peak) for each ion described by its mass/charge (m/z) and retention time (with the integration of its isotope envelope) were estimated using the Progenesis QI data analysis software (Nonlinear Dynamics). Abundances of detected lipid ions were normalized using the LPC(17:1) and PC(17:0-14:1) standards. Cation adducts (i.e., [M + Na]+ and [M + K]+) were combined with protonated ions ([M + H]+) before further statistical analysis. To confirm lipid class and length of fatty acyl chains selected ions were analyzed by LC–MS/MS in a separate run; fragmentation patterns were verified using SimLipid software (PREMIER Biosoft).

### Statistical and bioinformatic analyses

To detect differences between pre-RT samples of patients with more/less advanced cancer or different cancer location, we used the Mann–Whitney test (the majority of analyzed lipid levels had not normal distributions). As an adjustment for multiple testing, we used Benjamini–Hochberg correction; differences were considered significant when q‑value ≤ 0.05. The area under a BED–volume curve (i.e., the total absorbed radiation energy) was measured by trapezoidal rule for each sample. To calculate the volume of irradiated tissue for every 10 Gy of DVH, the spline regression model was built on the BED and radiation volume values. The knots value for BED were selected based on the minimum value, 0.25, 0.5, and 0.75 quantiles, and the maximum value separately for each patient. A fitted model was used to predict radiation volumes for a chosen BED value (range between 10 and 100 Gy). Spearman’s correlation coefficient was computed to examine the correlation between the specific lipid level and selected radiation parameter: max. GTV dose, the total absorbed radiation energy, tissue volumes of selected isodoses (separately from 10 to 100 Gy), all expressed in BED, and ARS measured at a time point that corresponded to the blood sample collection. All analyses were performed using R statistical software package version 4.0.1. (R Foundation for Statistical Computing, http://www.r-project.org). A correlation was considered significant when its two-sided *p*-values ≤ 0.05 and |r| > 0.3 [22].

## Results

The analysis of serum lipidome was performed in a group of HNSCC patients treated with IMRT alone according to different treatment plans (Table 1). Using the liquid chromatography coupled with mass spectrometry (LC-MS) approach, based on the compound’s retention times and mass/charge (m/z) values, we detected and quantitated 151 choline-containing lipids (or their isomer groups), including 81 phosphatidylcholines (PCs), 12 lysophosphatidylcholines (LPCs), and 58 sphingomyelins (SMs); all detected lipids are listed in Supplementary Table S3. Further fragmentation patterns using tandem mass spectrometry additionally confirmed the specific identification of 43 lipids (lipid names were used in this case instead of lipid class and m/z identifier). The analysis was performed in serum samples collected consecutively every week of treatment (approximately 5 intra-RT samples per patient, 249 samples in total). The involved group of patients was rather heterogeneous concerning cancer stage and location (Table 1). Therefore, to estimate the potential effect of these confounding factors, relevant differences in lipid profiles were addressed in pre-RT serum samples. Only a few lipids were found that showed different levels in sera of patients with less and more advanced cancer. When compared patients with T1–T2 vs. T3–T4 tumor size and patients with N0 vs. N1–3 local lymph node status, there were 9 and 14 compounds that showed differences at the significance level *p* < 0.05, respectively, yet none of them remained statistically significant if the correction against multiple testing was applied. Similarly, no statistically significant differences were observed when pre-RT serum samples were compared for patients with two major cancer locations (i.e., larynx and pharynx). Therefore, we concluded that RT-related changes observed in analyzed samples should be primarily affected by doses of radiation and/or volume of irradiated tissues.

In general, we searched for the association between RT-induced changes in levels of specific phospholipids and radiation dose (or volume of irradiated tissues). However, the study included patients treated with different irradiation schemes with putatively different biological effects. Therefore, to compensate for this effect, biologically effective doses (BED) were calculated in each case and used instead of “physical” doses (Supplementary File Table S1). In the first step, we searched for lipids which serum levels were associated with a maximum GTV (gross tumor volume) dose accumulated at a given time point (not only at the end of RT). We found 16 lipids (2 LPCs, 11 PCs, and 3 SMs) that showed statistically significant correlations (*p*-value < 0.05 and |r| > 0.3) with a maximum GTV dose (examples of identified compounds are presented in Table 2). Furthermore, we searched for the association between levels of lipids and total radiation energy absorbed by the patient’s body. This parameter was estimated from the individual dose–volume histogram (DVH) by calculating its integral over a dose or area under the curve (radiation dose multiplied by mass approximated from a volume of irradiated tissue reflected absorbed energy). In this case, we found 27 lipids (2 LPCs, 22 PCs, and 3 SMs) whose serum levels were significantly correlated with total absorbed radiation energy accumulated in the patient’s body at a given time point (examples of identified compounds are presented in Table 2). Due to the characteristics of IMRT, tissue irradiated with high doses represent rather small volumes (tumor and adjacent tissues) and may have a lower impact on the systemic body’s response to RT observed at the level of body fluids. Hence, this latter observation suggested the importance of “lower” doses of radiation delivered to “larger” volumes of normal tissues.

**Table 2:** Examples of identified lipids which serum levels correlated with maximum GTV dose, a total absorbed radiation energy (i.e., area under DVH), volumes of tissues irradiated at 20 or 50 Gy of BED, and acute radiation toxicity. Showed are correlation coefficients; significant correlations (|r| > 0.3) are marked with bold

Lipid name	m/z	Max. GTV dose	Total absorbed energy	V20	V50	ARS
LPC(16:1)	494.33	−0.31	−0.32	−0.33	−0.16	−0.32
LPC(18:0)	524.38	**−0.39**	**−0.37**	**−0.35**	−0.21	−0.29
PC(30:0)	706.55	**−0.32**	**−0.34**	**−0.35**	−0.25	**−0.34**
PC(30:1)	704.53	**−0.31**	**−0.34**	**−0.35**	−0.20	**−0.33**
PC(32:2)	730.55	**−0.39**	**−0.37**	**−0.35**	−0.27	**−0.38**
PC(34:3)	756.57	**−0.33**	**−0.35**	**−0.35**	−0.28	−0.26
PC(36:2)	786.61	**−0.41**	**−0.43**	**−0.42**	−0.28	−0.28
PC(36:3)	784.60	**−0.30**	**−0.30**	−0.27	−0.10	−0.24
PC(38:2)	814.64	**−0.48**	**−0.51**	**−0.48**	**−0.33**	**−0.37**
PC(38:3)	812.63	**−0.49**	**−0.50**	**−0.46**	−0.28	**−0.38**
PC(38:5)	808.60	−0.29	**−0.34**	**−0.32**	−0.16	**−0.31**
SM(36:0)	733.63	**0.31**	**0.37**	**0.37**	0.26	**0.30**
SM(36:1)	731.62	**0.34**	**0.36**	**0.36**	0.25	0.25

*GTV* gross tumor volume, *DVH* dose–volume histogram, *BED* biologically effective doses, *V20* volumes irradiated with at least 20 Gy of BED, *V50* volumes irradiated with at least 50 Gy of BED, *m/z* mass to charge ratio, *ARS* acute radiation sequel, *LPC* lysophosphatidylcholine, *PC* phosphatidylcholine, *SM* sphingomyelin. Listed lipids represent groups of isomers that cannot be separated using the implemented LC-MS approach. Names of compounds reflect numbers of carbon atoms and double bonds in fatty acid residues

**Fig. 1:**
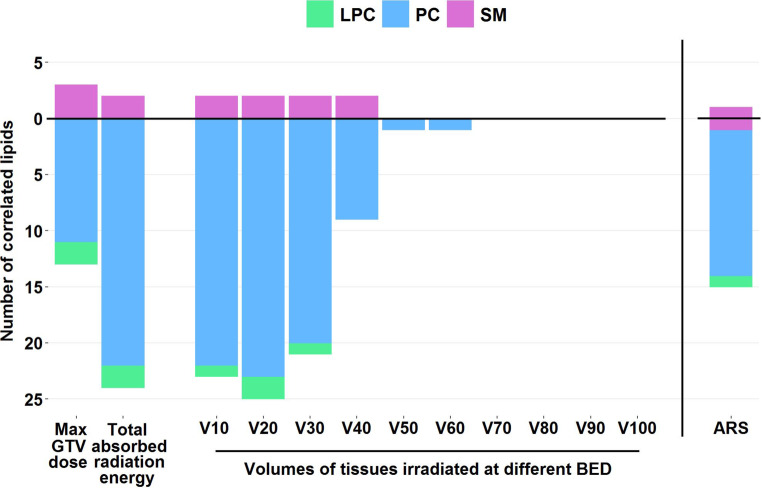
Numbers of lipids which serum levels were significantly correlated with maximum GTV dose, a total absorbed radiation energy, and volumes of tissues irradiated at different BED (10 Gy intervals in the 10 to 100 Gy range). Different classes of lipids (LPC, PC, and SM) are color-coded; positive (r > 0.3) and negative (r < −0.3) correlations are presented *above* and *below the zero line*, accordingly. *ARS* acute radiation sequel, *GTV* gross tumor volume, *BED* biologically effective doses, *LPC* lysophosphatidylcholine, *PC* phosphatidylcholine, *SM* sphingomyelin

To study this effect in detail, we extracted from each DVH (modeled both at the end of RT and for each time point during RT) the irradiated tissue volumes for every 10 Gy increase of accumulated BED from 10–100 Gy. Then, lipids which serum levels correlated with a volume of tissues irradiated at a given BED were identified. We found the highest number of such correlations with the volume of tissues irradiated at 20 Gy BED (27 correlated compounds, examples of identified compounds are presented in Table 2). The number of compounds whose serum levels were correlated with a volume of irradiated tissues gradually decreased with a dose. Therefore, practically no lipids were detected whose serum levels correlated significantly with volumes of tissues irradiated at BED higher than 40 Gy (Fig. 1).

**Fig. 2:**
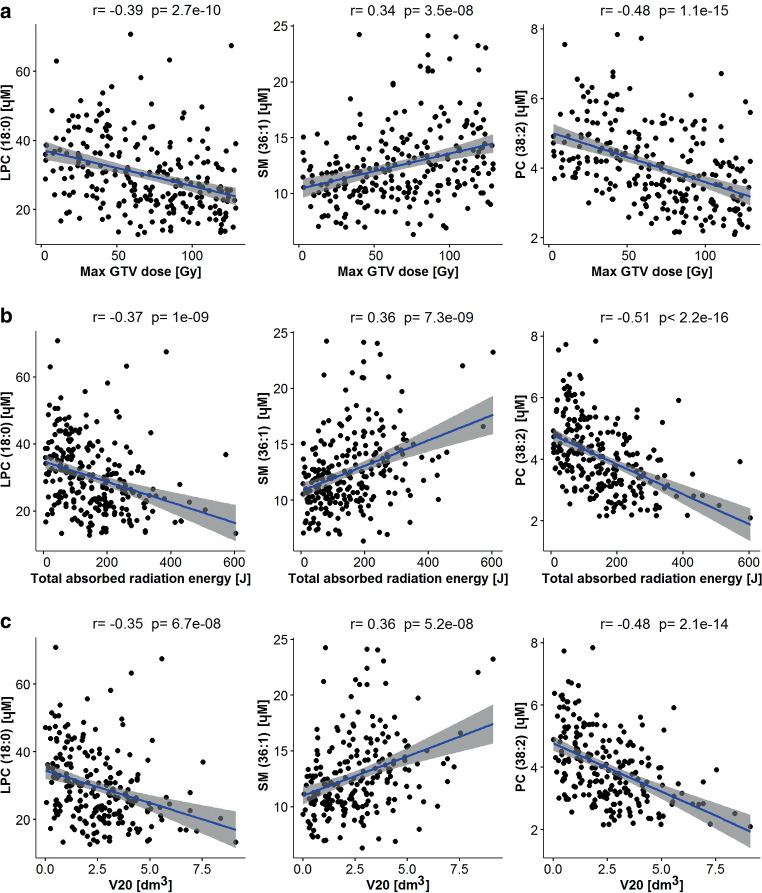
Correlations between serum levels of selected lipids and maximum GTV dose (**a**), the total absorbed radiation energy (**b**), and volume of tissues irradiated at 20 Gy BED (**c**). Illustrated are three compounds: LPC(18:0), SM(36:1), and PC(38:2). *Grey zone* represents 95% confidence intervals for the correlation coefficient. *Max GTV* maximum gross tumor volume, *BED* biologically effective doses, *V20* volumes irradiated with at least 20 Gy of BED, *LPC* lysophosphatidylcholine, *PC* phosphatidylcholine, *SM* sphingomyelin

We found different types of correlations with dose/volume for specific types of lipids. In general, the increased dose or volume of irradiated tissues that was associated with decreased serum lipid level (i.e., negative correlation) was observed for several PCs and LPCs. Changes in serum levels of about a third part of detected PCs showed some correlation with radiation parameters. For example, a negative correlation was observed between levels of 25 PCs (out of 81) and volume of tissues irradiated at 20 Gy BED; this class of lipids is exemplified by PC(38:2) as illustrated in Fig. 2. Similarly, negative correlations with radiation parameters were observed for 2 LPCs (out of 12 detected), namely LPC(16:1) and LPC(18:0); the latter one is illustrated in Fig. 2. Noteworthy, positive correlations were observed for neither PCs nor LPCs. In marked contrast, for 3 SMs (out 51 detected) the increased serum levels were associated with increased dose or volume of irradiated tissues (i.e., positive correlations); this class of lipids is exemplified by SM(36:1) as illustrated in Fig. 2. No negative correlations with radiation parameters were observed for SMs, which indicated different modes of reactivity of SMs and PCs/LPCs.

**Fig. 3:**
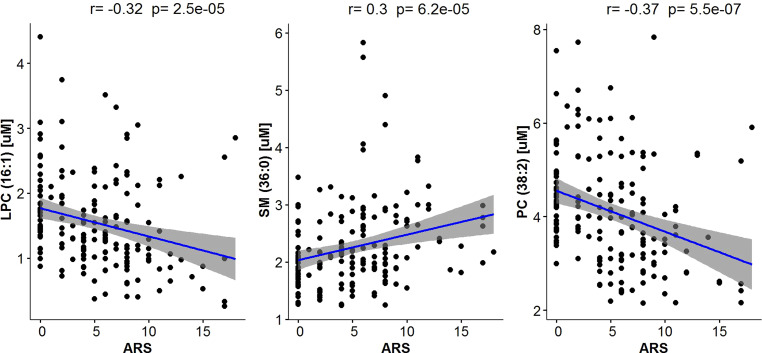
Correlations between serum levels of selected lipids and acute radiation toxicity. Illustrated are three compounds: LPC(16:1), SM(36:0), and PC(38:2). *ARS* acute radiation sequel, *LPC* lysophosphatidylcholine, *PC* phosphatidylcholine, *SM* sphingomyelin

Furthermore, we searched for possible associations between serum lipid levels and the degree of the acute radiation response (acute radiation sequel; ARS) noted at the time of blood sample collection. Several phospholipids were significantly correlated with ARS scores (Table 1, Fig. 1). Levels of 13 PCs showed negative correlations with ARS scores, which is exemplified by PC(38:2) in Fig. 3. Moreover, levels of LPC(16:1) showed a negative correlation, while levels of SM(36:0) showed a positive correlation with ARS scores, which is illustrated in Fig. 3. Hence, serum lipid profiles were similarly affected by acute radiation toxicity and the dose of radiation, which putatively reflected a close association between radiation dose (or irradiated volume) and acute radiation response.

## Discussion

Several bioindicators of exposure to ionizing radiation have been proposed, yet the actual dose-dependence in samples of human tissues exposed to radiation in vivo was documented for only a few of them. Such verified “biodosimeters” include factors that could be tested in peripheral blood cells: (cyto)genetic lesions [23] and the expression of certain genes like *FXDR* [24]. Here we documented the dose and volume dependence of RT-induced changes in a profile of human serum phospholipids, which further extend the pool of potential biomarkers of radiation exposure. We observed a dose-dependent decrease of (lyso)phosphatidylcholines (LPCs/PCs) and increase of sphingomyelins (SMs) in the serum of patients exposed to partial body irradiation due to head and neck cancer. Moreover, a similar pattern of relationships between levels of serum lipids and the escalation of acute radiation response was observed, which putatively reflected a close link between acute toxicity and radiation doses or volumes of irradiated tissues. Importantly, observed effects on lipid profiles were associated not only with a maximum radiation dose delivered to the gross tumor volume but also with lower doses delivered to larger volumes of normal tissues (assuming the dose deposition proportional to the total/maximum dose, for 20 Gy BED these may represent approximately 0.5 Gy per fraction). In fact, high doses linked with small volumes (e.g., max. GTV dose) induced markedly lower effects in serum lipid profiles than lower doses linked with high volumes of irradiated tissues. We had previously found that the extent of differences in profiles of endogenous serum peptides [10] and lipids [18] between pre-RT/within-RT and post-RT samples of HNC patients treated with a continuous accelerated RT (CAIR) correlated mainly with the volume of tissues irradiated at relatively low doses (accumulated dose 10–15 Gy that corresponded to a fraction dose 0.3–0.5 Gy). Here we extended this observation for other schemes of IMRT and focused on specific classes of phospholipids. Interestingly, our observations may fit into the concept of the low dose hyperradiosensitivity (HRS), which assumes that low doses (< 0.5 Gy) are more effective in cell killing than high doses per unit dose [25].

In this work, we addressed choline-containing phospholipids present in human serum. Phosphatidylcholines, the main building blocks of membrane bilayers, are the most abundant phospholipids in serum/plasma that are predominantly located in high-density lipoproteins (HDL). Decreased levels of PCs in the serum of irradiated patients putatively reflect their rapid turnover in stressed/damaged cells and elevated uptake from the blood. In addition to their main function as a membrane constituent, PCs have a role in signaling through the generation of LPCs (by phospholipases A2), SMs (by SM synthase), phosphatidic acid (PA; by phospholipases D), and diacylglycerols (DAG; by phospholipases C) [26]. Therefore, significant downregulation of several PCs observed in serum during radiotherapy could reflect both the recovery of the damaged cell membrane and increased requirements for signaling pathways that depend on PC-derived compounds. Lysophosphatidylcholines are the major bioactive component of oxidized low-density lipoproteins (LDL) mostly responsible for their inflammation-related functions [27]. Decreased levels of LPCs in blood were significantly associated with activated inflammatory status in many cancer types [28], which suggest that the reduced level of LPCs observed in serum of irradiated patients could also reflect the inflammation-related aspect of radiation response. In contrast to PCs and LPCs, in which serum levels decreased during radiotherapy, sphingomyelins showed radiation-related upregulation. In general, choline-based compounds are constantly transformed into each other [14]. Hence, new SM molecules are likely generated from PC compounds by SM synthase, which transfers choline “head” from PC to suitable ceramide molecule, which also explains the reduced level of ceramides observed in serum of irradiated patients [18]. SMs can be hydrolyzed back to ceramides by SMase action. This balance between sphingomyelin production and degradation is a key factor in SM-related apoptotic signaling, and the generation of ceramides from SMs’ degradation was reported to influence both the rate and the form of cell death [29]. Therefore, observed changes in serum levels of choline-containing phospholipids apparently mirror the membrane regeneration processes and signal-transduction pathways associated with treatment-induced damage of cellular and tissue components.

Interestingly, RT-downregulated LPC that is based on stearic acid (18:0) is directly produced through phospholipase A2 from PCs that contain this fatty acid. PCs that putatively contain stearic acid, namely PC(18:0/18:2), PC(18:0/18:3), PC(18:0/20:2), PC(18:0/20:3), and PC(18:0/20:5) (detected as PC 36:2, 36:3, 38:2, 38:3, and 38:5, respectively) were also downregulated by RT. Furthermore, stearic acid was a component of RT-upregulated SMs, namely SM(d18:0/18:0) and SM(d18:1/18:0) (detected as SM 36:0 and 36:1, respectively). Therefore, our observations indicate that the metabolism of stearic acid is particularly involved in radiation response. The linkage between stearic acid and choline-containing phospholipids as well as their radiation-related functions are complicated and might also be affected by other processes ongoing in the patient’s body. Nevertheless, these compounds present in human serum or plasma could be used to monitor systemic effects induced by ionizing radiation with some potential as bioindicators of radiation exposure.

The IMRT techniques leading in contemporary radiation oncology enable the delivery of prescribed doses in the target areas not exceeding the tolerance doses for healthy organs. However, full protection of healthy tissues is practically impossible to implement with the use of dynamic photon radiotherapy (i.e., IMRT) because of its primary characteristics (i.e., buildup region and loss of energy with depth), which is further magnified by the use of a large number of treatment fields from different directions. Assuming that disease recurrence is observed in 30–50% of HNSCC patients, the appropriate dose distribution in the cancer target is the obvious priority nowadays. However, the importance of biological effects associated with “clinically” low doses absorbed in large volumes is increasingly raised, pointing to some shortcomings of IMRT. In the future, biomarkers of a systemic response to radiation may have clinical application in comparing the safety of photon-based techniques with particle-based techniques (e.g., proton therapy), which minimize the absorption of radiation doses in healthy tissues adjacent to cancer. Hence, the data provided in this manuscript both indicate hypothetical “biodosimetry” potential of serum lipid patterns and may contribute to the understanding of general mechanisms induced in the patient’s organism in response to partial body irradiation.

## Conclusions

This study demonstrates the significant involvement of phospholipids based on choline head and stearic acid residue in the systemic response of the patient’s body to IMRT. The serum levels of these compounds depended on the dose and volume of irradiated tissues. Higher doses and volumes were associated with reduced levels of (lyso)phosphatidylcholines and increased levels of sphingomyelins. Presented data suggest the “biodosimetry” potential of serum lipid patterns and indicate biological effects associated with low/moderate doses delivered to large volumes of normal tissue.

## Supplementary Information

Examples of recalculation of physical doses to BED are presented in the Supplementary File Table S1. The descriptive statistics of radiation parameters are presented in the Supplementary File Table S2. All detected lipids are listed in Supplementary Table S3.
